# Quantitative evaluation of the myocardial bridge anatomical features and FFR_CT_ in patients with myocardial bridging stratified by age

**DOI:** 10.1097/MD.0000000000045686

**Published:** 2025-11-07

**Authors:** Mengya Li, Fuqian Guo, Yafei Huang, Dan Zhang, Haowen Zhang, Caiying Li

**Affiliations:** a Department of Medical Imaging, The Second Hospital of Hebei Medical University, Shijiazhuang, China; b Department of Radiology, The Third Hospital of Hebei Medical University, Shijiazhuang, China.

**Keywords:** age, coronary computed tomography angiography, fractional flow reserve, left anterior descending coronary artery, myocardial bridging

## Abstract

Myocardial bridge (MB) is a congenital coronary artery anomaly associated with various cardiac events. This study aimed to evaluate the effect of age on the anatomical features and computed tomography-derived fractional flow reserve (FFR_CT_) of the left anterior descending coronary artery (LAD) MB by using coronary computed tomography angiography. A retrospective study of 139 patients with LAD MB was conducted by dividing patients into 2 groups based on MB length: the short MB group (<20 mm, n = 58) and the long MB group (≥20 mm, n = 81). Patients were further categorized into 3 age groups: young (<45 years, n = 28), middle-aged (45–59 years, n = 89), and elderly (≥60 years, n = 22) groups. Coronary computed tomography angiography was used to measure the anatomical features of the MB. FFR_CT_ values were calculated using a deep learning software at 3 locations along the LAD in the systolic and diastolic phases. Statistical analyses were performed using SPSS 21.0. In the long MB group, the FFR_CT_ values were lower than those in the short MB group; moreover, the △FFR in the long MB group was larger than that in the short MB group (*P* < .05). No significant differences were found in clinical data, MB anatomical features, or FFR_CT_ values among the age groups. In the middle-aged group, patients with abnormal FFR_CT_ values had a significantly longer MB, closer MB location, and higher muscle index than those with normal FFR_CT_ values (*P* < .05). In the elderly group, only MB length was significantly longer in patients with abnormal FFR_CT_ values (*P* < .05). The youth group showed no significant difference between the normal and abnormal FFR_CT_ groups. While age did not significantly affect MB anatomy and FFR_CT_ values overall, specific MB anatomical parameters may contribute to decreased FFR_CT_ values in middle-aged and older patients, providing valuable insights for MB hemodynamic assessment.

## 1. Introduction

The myocardial bridge (MB) is a congenital coronary artery anomaly that is increasingly associated with various adverse events. It can cause coronary artery spasm, tortuosity, and atherosclerosis, thereby affecting myocardial perfusion and coronary hemodynamics.^[1–6]^ In 2013, Taylor first proposed the concept of coronary computed tomography-derived fractional flow reserve (FFR_CT_).^[7]^ Due to its noninvasive nature and high consistency with angiography-derived FFR, FFR_CT_ has been widely utilized for measuring coronary blood flow reserve. While recent research has primarily focused on studying abnormalities in coronary blood flow reserves due to coronary atherosclerosis,^[8,9]^ some researchers have also explored the application of FFR_CT_ in the context of MB. However, limited research exists on the impact of age on anatomical features and FFR_CT_ of MB.

This study aims to evaluate the effect of age on the anatomical features and FFR_CT_ of the left anterior descending coronary artery (LAD) MB based on coronary computed tomography angiography (CCTA).

## 2. Methods

### 2.1. Clinical data

**Figure 1.**
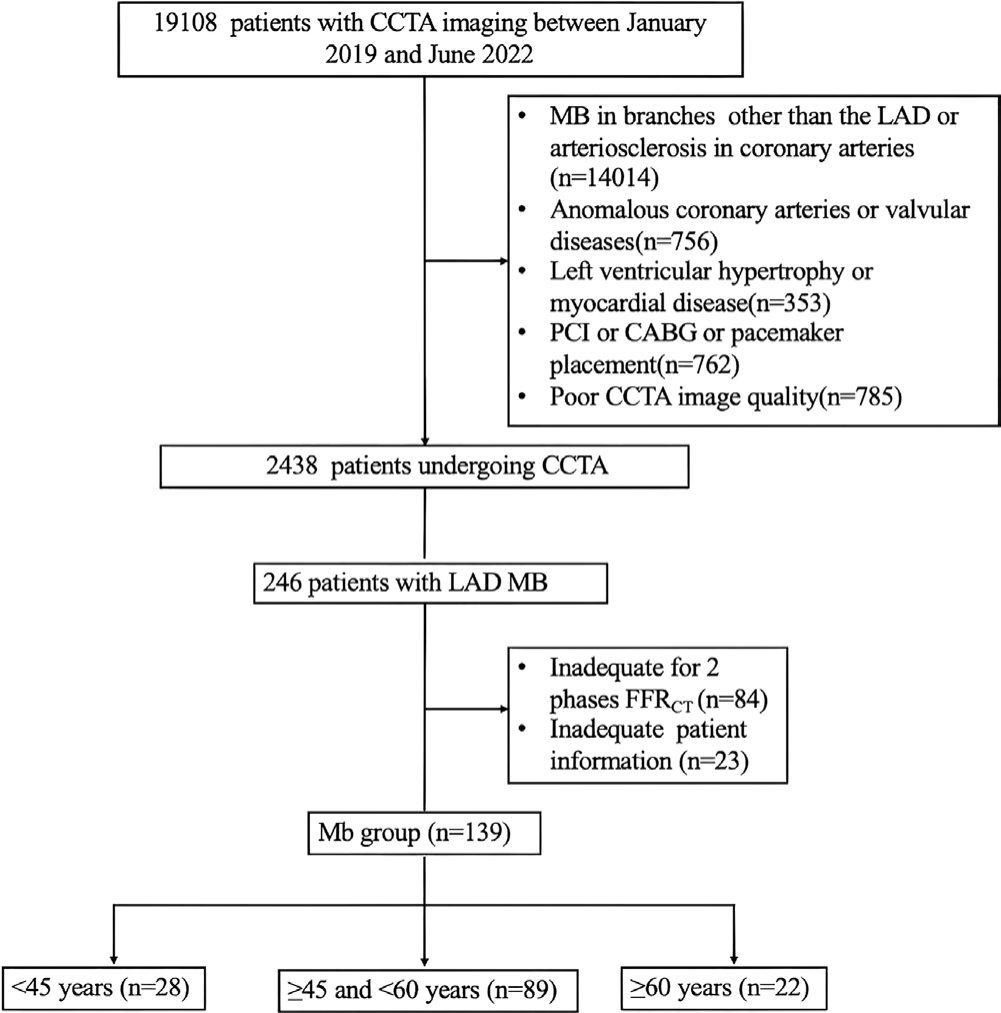
Flow chart of the study. CCTA = coronary computed tomography angiography, FFR_CT_ = CT-derived fractional flow reserve, LAD = left anterior descending artery, MB = myocardial bridging.

We retrospectively collected data from 139 patients who underwent CCTA examination at our hospital between January 2019 and June 2022 and were diagnosed with MB in the LAD. This study was approved by the Ethics Committee of the Second Hospital of Hebei Medical University (No. 2023-R430). A flowchart of the process is shown in Figure 1. Subjects were stratified into 2 groups based on MB length: the short MB group (<20 mm, n = 58) and the long MB group (≥20 mm, n = 81). Additionally, the patients were divided into 3 age groups: young (<45 years, 28 cases, 38.9 ± 3.6 years), middle-aged (≥45 and <60 years, 89 cases, 52.7 ± 3.7 years), and elderly (≥60 years, 22 cases, 63.7 ± 2.9 years). The inclusion criteria were as follows: inpatients or outpatients who underwent CCTA examination; clear imaging data with good contrast agent filling in the coronary arteries; and consent to participate in this study. The exclusion criteria were the presence of any coronary artery plaque (calcified or noncalcified), abnormal origin or termination of coronary arteries, other coronary artery diseases, such as coronary aneurysms, left ventricular hypertrophy, cardiomyopathy, heart valve disease, history of coronary intervention or bypass surgery, and missing or poor-quality images in any systolic or diastolic phase. General clinical data were recorded for all patients.

### 2.2. CCTA scanning protocol

All patients were scanned using a Philips 256-slice iCT scanner (Brilliance iCT; Philips Healthcare). Retrospective electrocardiogram-gated scanning was performed. After determining the scanning range via a positioning scan, a Stellant dual-barrel high-pressure injector was used to inject the nonionic contrast agent iohexol (350 mgI/mL) at a rate of 4 to -5 mL/s, with a dosage of 0.8 mL/kg, followed by an equal injection rate of 30 mL of 0.9% sodium chloride solution. An intelligent triggering technique was used, with the region of interest placed in the ascending aorta at the aortic window level, triggering threshold set at 150 HU, and a delay time of 5 seconds. Computed tomography scanning parameters were as follows: tube voltage, 120 kV; tube current, using automatic milliampere technology; collimator, 128 × 0.625 mm; tube rotation time 0.27 s/rotation, matrix 512 × 512. The image reconstruction phases were set at 45% and 75% of the cardiac cycle.

### 2.3. Image analysis

**Figure 2.**
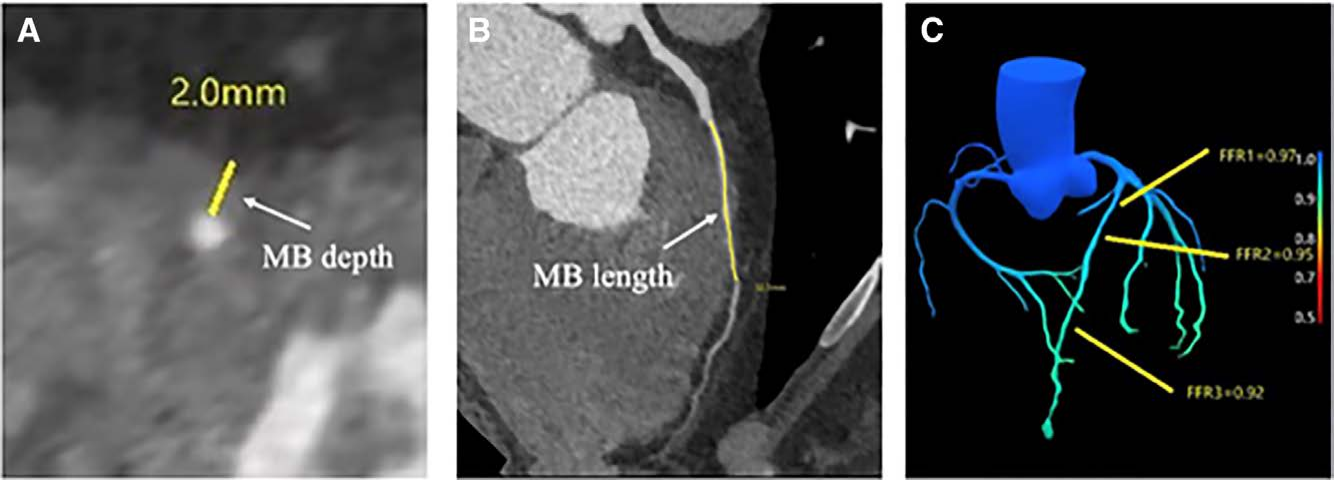
CCTA and FFR_CT_ features of the left anterior descending coronary artery with myocardial bridging. Deep MB in the mid-segment of the LAD in a 51-year-old man. (A) The MB depth is 2.0 mm. (B) The MB length is 32.3 mm. (C) The FFR_CT_ values distal to the MB (FFR3) is 0.92. CCTA = coronary computed tomography angiography, FFR_CT_ = CT-derived fractional flow reserve, LAD = left anterior descending coronary artery, MB = myocardial bridging.

CCTA data of all included patients were transferred to the Philips EBW 4.5 post-processing workstation (Brilliance workspace, Philips Healthcare, Amsterdam, Netherlands) for image analysis. The anatomical parameters of the MB were measured by a radiologist with 10 years of CCTA post-processing experience. The parameters included the MB depth, length, location, MB muscle index, and systolic compression. MB depth was defined as the maximum thickness of the myocardium covering the coronary artery, recorded as 1 mm when the maximum thickness was ≤ 1 mm (Fig. 2A)^[10]^; MB length was defined as the distance from the entrance to the exit of the MB (Fig. 2B); MB location was defined as the distance from the opening of the LAD to the entrance of the MB; muscle index was defined as MB depth × MB length^[11]^; systolic compression was defined as [(diastolic diameter ‐ systolic diameter)/diastolic diameter].^[12]^

The Shukun software (FFR V1.7, ShuKun Technology Co., Ltd., Beijing, China) was used to calculate the FFR_CT_ values using a deep learning algorithm. The FFR_CT_ values were measured at 3 locations (1 cm before the entrance of the MB, the middle segment of the MB, and 2–4 cm after the exit of the MB) in 2 phases (systole and diastole),^[13]^ recorded as FFR1, FFR2, and FFR3, respectively (Fig. 2C), with ΔFFR calculated as FFR1–FFR3. In the control group, FFR_CT_ values were measured at three specific points along the corresponding sites in the middle segment of the LAD. The software automatically calculated the FFR_CT_ value of the LAD in each phase, with an FFR_CT_ value ≤ 0.8 in any phase considered abnormal.^[14]^

### 2.4. Statistical analysis

SPSS software (version 21.0; IBM Corp., Armonk) was used for statistical analysis. Quantitative data conforming to a normal distribution were expressed as mean ± standard deviation, and comparisons between 2 groups were performed using the *t* test. Non-normally distributed quantitative data are expressed as medians with interquartile ranges, with comparisons between 2 groups using the Mann–Whitney *U* test and comparisons between 3 groups using the Kruskal–Wallis *H* test. Differences between categorical data groups were compared using the chi-square test. Statistical significance was set at *P* < .05.

## 3. Result

### 3.1. Patient characteristics

Clinical data, MB features, and FFR_CT_ values stratified by age are presented in Table 1. There were no significant differences in clinical data, MB anatomical features, or FFR_CT_ values at the three sites, as well as in △FFR among MB patients across the different age groups (*P* > .05).

**Table 1:** Comparison of clinical data, anatomical characteristics of myocardial bridge, and FFR_CT_ values among myocardial bridge patients of different ages.

Variable	<45 yr (n = 28)	≥45 and < 60 yr (n = 89)	≥60 yr (n = 22)	*P*-value
Demographics				
Gender (male), n (%)	19 (67.9)	48 (53.9)	9 (40.9)	.160
BMI (kg/m^2^)	25.8 (21.7, 27.4)	24.7 (23.4, 26.2)	23.4 (21.5, 25.1)	.102
Hypertension, n (%)	4 (14.3)	25 (28.1)	4 (18.2)	.261
Hyperlipidemia, n (%)	1 (3.6)	9 (10.1)	3 (13.6)	.440
Diabetes, n (%)	3 (10.7)	5 (5.6)	4 (18.2)	.128
Angina status				
Typical, n (%)	3 (10.7)	8 (9.0)	2 (9.1)	.915
Atypical, n (%)	0 (0.0)	5 (5.6)	1 (4.5)	.495
Chest tightness and pain, n (%)	4 (14.3)	6 (6.7)	4 (18.2)	.183
None, n (%)	21 (75)	70 (78.7)	15 (68.2)	.593
MB characteristics				
MB depth, mm	1.3 (1.0, 3.6)	1.6 (1.0, 3.1)	1.1 (1.0, 2.7)	.804
MB length, mm	26.5 (16.8, 34.8)	22.0 (16.2, 30.7)	17.5 (14.1, 26.5)	.146
MB location, mm	30.9 (29.4, 49.3)	30.9 (29.4, 49.3)	31.9 (26.6, 44.0)	.781
MB muscle index	36.0 (20.0, 114.9)	34.6 (20.3, 76.9)	30.0 (14.6, 87.8)	.471
Systolic compression index, %	10.6 (1.3, 21.7)	7.7 (5.3, 13.6)	9.1 (5.7, 18.1)	.412
FFR_CT_ features				
Systolic phases				
FFR1	1.00 (1.00, 1.00)	1.00 (1.00, 1.00)	1.00 (1.00, 1.00)	.659
FFR2	0.99 (0.97, 1.00)	0.99 (0.98, 1.00)	1.00 (0.96, 1.00)	.507
FFR3	0.91 (0.84, 0.97)	0.93 (0.89, 0.97)	0.95 (0.91, 0.97)	.258
△FFR	0.10 (0.03, 0.15)	0.07 (0.03, 0.11)	0.04 (0.02, 0.08)	.072
Diastolic phases				
FFR1	1.00 (1.00, 1.00)	1.00 (1.00, 1.00)	1.00 (1.00, 1.00)	.076
FFR2	0.99 (0.96, 1.00)	0.99 (0.97, 1.00)	1.00 (0.99, 1.00)	.270
FFR3	0.91 (0.86, 0.96)	0.93 (0.89, 0.97)	0.95 (0.87, 0.98)	.342
△FFR	0.08 (0.03, 0.14)	0.07 (0.03, 0.11)	0.05 (0.02, 0.13)	.513

FFR_CT_ = CT-derived fractional flow reserve, MB = myocardial bridging.

### 3.2. Comparison of FFR_CT_ values at different sites between the short myocardial bridge group (<20 mm) and the long myocardial bridge group (≥20 mm)

No significant intergroup difference in FFR1 was observed (*P* > .05).FFR2 and FFR3 values at the mid and distal segments were significantly lower in the long MB group compared to the short MB group. The △FFR exhibited a significantly greater magnitude in the long MB group (*P* < .05) (Table 2).

**Table 2:** Comparison of FFR_CT_ values at different sites between the short myocardial bridge group (<20 mm) and the long myocardial bridge group (≥20 mm).

Variables	Short MB (<20 mm) (n = 58)	Long MB (≥20 mm) (n = 81)	*P*-value
Systolic phases			
FFR1	1.00 (1.00, 1.00)	1.00 (1.00, 1.00)	.514
FFR2	1.00 (0.99, 1.00)	0.99 (0.97, 1.00)	.001
FFR3	0.96 (0.89, 0.98)	0.91 (0.86, 0.95)	.006
△FFR	0.04 (0.02, 0.09)	0.08 (0.04, 0.13)	.003
Diastolic phases			
FFR1	1.00 (1.00, 1.00)	1.00 (1.00, 1.00)	.977
FFR2	0.99 (0.99, 1.00)	0.99 (0.96, 1.00)	.003
FFR3	0.95 (0.91, 0.98)	0.91 (0.86, 0.96)	.003
△FFR	0.05 (0.02, 0.09)	0.08 (0.04, 0.14)	.004

FFR_CT_ = CT-derived fractional flow reserve, MB = myocardial bridging.

### 3.3. Comparison of MB anatomical features between patients with normal and abnormal FFR_CT_ across different age groups

In the youth group (<45 years), the analysis revealed no statistical differences in the various anatomical features of the MB between patients with normal and abnormal FFR_CT_ values (*P* > .05) (Table 3).

**Table 3:** Comparison of anatomical characteristics of myocardial bridge between patients with abnormal and normal FFR_CT_ values in the youth group (<45 yr).

Variables	Abnormal FFR_CT_ (n = 16)	Normal FFR_CT_ (n = 12)	*P*-value
MB depth, mm	1.1 (1.0, 3.8)	2.5 (1.0, 3.6)	.439
MB length, mm	25.6 ± 10.8	28.5 ± 10.3	.478
MB location, mm	31.8 (29.6, 53.8)	30.8 (28.3, 43.6)	.642
MB muscle index	28.8 (18.6, 100.1)	69.9 (25.1, 114.9)	.378
Systolic compression index, %	12.1 (1.3, 23.3)	7.5 (1.4, 13.0)	.283

FFR_CT_ = CT-derived fractional flow reserve, MB = myocardial bridging.

In the middle-aged group (≥45 and <60 years), patients with abnormal FFR_CT_ values had a significantly longer MB, closer MB location, and higher muscle index than those with normal FFR_CT_ values (*P* < .05). However, there were no statistically significant differences in the MB depth and systolic compression between the 2 groups (*P* > .05) (Table 4).

**Table 4:** Comparison of anatomical characteristics of myocardial bridge between patients with abnormal and normal FFR_CT_ values in the middle-aged group (≥45 and < 60 yr).

Variables	Abnormal FFR_CT_ (n = 40)	Normal FFR_CT_ (n = 49)	*P*-value
MB depth, mm	1.9 (1.0, 3.1)	1.2 (1.0, 3.3)	.200
MB length, mm	24.9 (18.9, 35.5)	19.1 (12.6, 28.2)	.003
MB location, mm	30.7 (27.0, 89.5)	39.3 (28.4, 49.4)	.041
MB muscle index	45.1 (27.8, 89.5)	28.4 (14.7, 62.5)	.006
Systolic compression index, %	8.3 (1.3, 14.1)	7.1 (5.3, 13.2)	.562

FFR_CT_ = CT-derived fractional flow reserve, MB = myocardial bridging.

In the elderly group (≥60 years), the results indicated that the MB length was significantly longer in the group with abnormal FFR_CT_ values than in the group with normal FFR_CT_ values (*P* < .05). No statistical differences were observed in the other anatomical parameters of the MB between the 2 groups (*P* > .05) (Table 5).

**Table 5:** Comparison of anatomical characteristics of myocardial bridge between patients with abnormal and normal FFR_CT_ values in the elderly group (≥60 yr).

Variables	Abnormal FFR_CT_ (n = 7)	Normal FFR_CT_ (n = 15)	*P*-value
MB depth, mm	1.0 (1.0, 1.8)	1.0 (1.0, 1.0)	.624
MB length, mm	25.7 (18.2, 54.0)	15.1 (13.5, 21.6)	.011
MB location, mm	33.9 ± 12.0	36.1 ± 9.8	.659
MB muscle index	87.4 (23.5, 99.6)	23.6 (13.7, 39.1)	.098
Systolic compression index, %	14.3 (3.1, 18.7)	9.1 (6.1, 16.9)	.944

FFR_CT_ = CT-derived fractional flow reserve, MB = myocardial bridging.

## 4. Discussion

MB is an anatomical variant that is increasingly linked to various clinical manifestations such as angina, myocardial infarction, arrhythmia, and sudden cardiac death.^[15–18]^ MB is most commonly found in the mid-segment of the LAD artery (57%), followed by the distal segment (15%).^[19]^ This study focused on patients with single-vessel disease located in the LAD MB, undertaking a detailed quantitative analysis of MB’s anatomical and functional parameters of the MB.

Before the widespread use of CCTA, MB was mostly discovered incidentally during coronary angiography (CAG). While CAG cannot directly detect MB, it can demonstrate the “milking effect” through various projection angles, revealing significant stenosis of the intramural coronary artery during systole, which disappears or reduces during diastole, aiding in diagnosis. However, most intramural coronary arteries do not show significant systolic narrowing during CAG, lacking the typical “milking effect,” which results in a detection rate of only 2% to 6%.^[20]^

CCTA has become the most commonly used diagnostic method for MB. CCTA can collect three-dimensional data and reconstruct images via post-processing workstation, clearly illustrating anatomical features and the relationship between MB and the intramural coronary artery. By measuring the diameter of the intramural coronary artery during systole and diastole, CCTA can also calculate the systolic compression index. CCTA is favored due to its high detection rate and accuracy, low radiation dose, low cost, and operational simplicity.

FFR_CT_, based on raw CCTA data, can quantitatively assess myocardial ischemia. Studies have confirmed FFR_CT_’s high accuracy in diagnosing myocardial ischemia, with strong consistency with catheter-based FFR, offering a noninvasive supplement to CCTA.^[21,22]^ After years of development, FFR_CT_ has demonstrated significant clinical value in diagnosing and treating coronary artery disease. Its application has expanded beyond stable coronary artery disease to acute chest pain cases, confirming its feasibility in emergency settings.^[23]^

In recent years, some researchers have applied FFR_CT_ to the hemodynamic study of MB. Zhou et al indicated that FFR_CT_ values decreased in the mid and distal segments of the MB, with the depth and length of the MB being risk factors for the decrease in FFR_CT_ values.^[13]^ Other studies found no statistical difference in MB length between groups with abnormal and normal FFR_CT_ values. It appears that MB length and location, more so than MB depth, are significant factors in hemodynamic changes.^[5]^ Our previous research has shown that both isolated MB and MB combined with atherosclerosis can lead to abnormal FFR_CT_ values. For patients without atherosclerosis, MB length is a crucial factor affecting FFR_CT_, whereas in cases of MB with atherosclerosis, the degree of stenosis in the LAD becomes the primary influencing factor.^[24]^ In this study, we selected patients with isolated MB involving a single lesion in the LAD. Based on a cutoff value of 20 mm, MBs were categorized into short MB (<20 mm) and long MB (≥20 mm) groups. The results showed that the longer the MB, the greater its impact on FFR_CT_ values, further confirming the correlation between MB length and FFR_CT_.

Researches show that the prevalence of coronary artery disease increases with age, with older patients more prone to developing atrial fibrillation, acute coronary syndrome, and other conditions.^[25,26]^ However, there is limited research on age’s impact on MB anatomy and hemodynamics. This study, using the 2023 World Health Organization age standards, dividing patients into youth (<45 years), middle-aged (≥45 and <60 years), and elderly (≥60 years) groups to assess the effect of age on the anatomical features and FFR_CT_ of LAD MB. The study found no differences in general clinical data among MB patients of different ages, suggesting that MB is a congenital developmental anomaly. In patients with LAD MB without coronary artery disease, age does not significantly affect clinical data. Further hemodynamic indicator comparisons revealed no significant differences in MB anatomy and FFR_CT_ values across different ages, indicating that age does not impact MB anatomy and FFR_CT_ values when MB is the sole condition.

The study further compared the anatomical features of MB between groups with abnormal and normal FFR_CT_ values across different age groups, finding that FFR_CT_ influencing factors vary with age. In patients under 45, MB anatomy does not affect coronary hemodynamics, possibly due to the heart’s strong reserve function and the absence of other coronary artery diseases. However, in middle-aged patients, MB anatomical parameters (length, location, and muscle compression index) can alter coronary hemodynamics, likely due to structural and endothelial changes in intramural coronary artery vessels, accelerated by direct myocardial compression and stimulation, leading to atherosclerosis and other conditions. The decline in heart reserve function with age reveals MB anatomy’s impact on coronary hemodynamics. In the elderly group (age ≥60 years), study findings indicated that MB length was the only factor influencing coronary hemodynamics. With increasing age, hypertrophy of vascular smooth muscle cells and expansion of the extracellular matrix lead to intimal thickening, increased stiffness, and reduced compliance,^[27]^ ultimately resulting in decreased vascular elasticity. Additionally, the renewal of myocardial cells slows significantly with age, leading to progressive senescence and apoptosis,^[28]^ which reduces myocardial contractility. Consequently, the compression exerted by the myocardial bridge on the mural coronary artery is reduced. These factors may explain why the correlation between muscle contraction index and abnormal FFR_CT_ values was weaker in the elderly group than in the middle-aged group. Coronary flow reserve is influenced by multiple factors. This study exclusively investigated the correlation between select MB anatomical parameters and abnormal FFR_CT_ values, and other unexamined parameters may also impact flow reserve, warranting further investigation.

This study quantitatively analyzed patients with LAD MB and found no significant impact of age on MB anatomy and coronary blood flow reserve among different age groups. However, different MB anatomical parameters were shown to cause coronary blood flow reserve reduction in different age groups. When patients are younger than 45 years, the anatomical characteristics of MB have no impact on FFR_CT_, likely due to better compensatory mechanisms in younger individuals. Parameters such as MB depth, length, location, muscle contraction index, and systolic stenosis rate exert minimal influence on FFR_CT_ values. As age increases (≥45 and <60 years), reduced vascular elasticity and decreased cardiac compensatory and reserve functions make MB length, location, and muscle contraction index contributors to abnormal FFR_CT_. In patients aged ≥60 years, further decline in vascular elasticity, cardiac compensatory and reserve functions, and myocardial contractility renders MB length the sole anatomical factor influencing abnormal FFR_CT_ values. Therefore, it is recommended that age be considered when conducting quantitative studies on MB hemodynamics.

The limitations of this study are as follows: its retrospective, single-center design, which may introduce selection bias; the absence of patient follow-up to assess clinical outcomes, highlighting the need for future research to better understand the impact of MB anatomical parameters and FFR_CT_ values on clinical outcomes.

## 5. Conclusions

This study initially explored the anatomical characteristics and FFR_CT_ values of the LAD MB in different age groups. The results showed no significant differences in clinical data, MB anatomy, or FFR_CT_ values among the various age groups. Nonetheless, in middle-aged and older patients, specific anatomical parameters of the MB may contribute to a decrease in FFR_CT_ values, thereby offering valuable clinical insight.

## Author contributions

**Conceptualization:** Caiying Li.

**Data curation:** Mengya Li, Fuqian Guo, Yafei Huang, Dan Zhang, Haowen Zhang.

**Formal analysis:** Mengya Li, Fuqian Guo, Yafei Huang.

**Investigation:** Mengya Li, Fuqian Guo, Yafei Huang.

**Methodology:** Dan Zhang, Haowen Zhang.

**Supervision:** Caiying Li.

**Writing – original draft:** Mengya Li.

**Writing – review & editing:** Fuqian Guo, Yafei Huang, Dan Zhang, Haowen Zhang, Caiying Li.
